# Zoonotic origins of human coronaviruses

**DOI:** 10.7150/ijbs.45472

**Published:** 2020-03-15

**Authors:** Zi-Wei Ye, Shuofeng Yuan, Kit-San Yuen, Sin-Yee Fung, Chi-Ping Chan, Dong-Yan Jin

**Affiliations:** 1 Department of Microbiology, The University of Hong Kong, Pokfulam, Hong Kong.; 2 School of Biomedical Sciences, The University of Hong Kong, Pokfulam, Hong Kong.

**Keywords:** coronavirus, SARS-CoV, SARS-CoV-2, MERS-CoV, COVID-19, animal reservoir, bats

## Abstract

Mutation and adaptation have driven the co-evolution of coronaviruses (CoVs) and their hosts, including human beings, for thousands of years. Before 2003, two human CoVs (HCoVs) were known to cause mild illness, such as common cold. The outbreaks of severe acute respiratory syndrome (SARS) and the Middle East respiratory syndrome (MERS) have flipped the coin to reveal how devastating and life-threatening an HCoV infection could be. The emergence of SARS-CoV-2 in central China at the end of 2019 has thrusted CoVs into the spotlight again and surprised us with its high transmissibility but reduced pathogenicity compared to its sister SARS-CoV. HCoV infection is a zoonosis and understanding the zoonotic origins of HCoVs would serve us well. Most HCoVs originated from bats where they are non-pathogenic. The intermediate reservoir hosts of some HCoVs are also known. Identifying the animal hosts has direct implications in the prevention of human diseases. Investigating CoV-host interactions in animals might also derive important insight on CoV pathogenesis in humans. In this review, we present an overview of the existing knowledge about the seven HCoVs, with a focus on the history of their discovery as well as their zoonotic origins and interspecies transmission. Importantly, we compare and contrast the different HCoVs from a perspective of virus evolution and genome recombination. The current CoV disease 2019 (COVID-19) epidemic is discussed in this context. In addition, the requirements for successful host switches and the implications of virus evolution on disease severity are also highlighted.

## Introduction

Coronaviruses (CoVs) belong to the family *Coronaviridae*, which comprises a group of enveloped, positive-sensed, single-stranded RNA viruses 1,2. These viruses harbouring the largest genome of 26 to 32 kilobases amongst RNA viruses were termed “CoVs” because of their crown-like morphology under electron microscope 2,3. Structurally, CoVs have non-segmented genomes that share a similar organization. Approximately two thirds of the genome contain two large overlapping open reading frames (ORF1a and ORF1b), which are translated into the pp1a and pp1ab replicase polyproteins. The polyproteins are further processed to generate 16 non-structural proteins, designated nsp1~16. The remaining portion of the genome contains ORFs for the structural proteins, including spike (S), envelope (E), membrane (M) and nucleoprotein (N). A number of lineage-specific accessory proteins are also encoded by different lineages of CoVs 2,4.

Based on the difference in protein sequences, CoVs are classified into four genera (alpha-CoV, beta-CoV, gamma-CoV and delta-CoV), among which the beta-CoV genera contains most HCoVs and is subdivided into four lineages (A, B, C and D) 2,4,5. Phylogenetic evidence has shown that bats and rodents serve as the gene source of most alpha-CoVs and beta-CoVs, while birds are the main reservoir of gamma-CoVs and delta-CoVs 2. For thousands of years, CoVs have constantly crossed species barriers and some have emerged as important human pathogens 2,4,6-8. To date, seven human CoVs (HCoVs) are known. Among them HCoV-229E and HCoV-NL63 are alpha-CoVs. The other five beta-CoVs include HCoV-OC43, HCoV-HKU1, severe acute respiratory syndrome coronavirus (SARS-CoV), Middle East respiratory syndrome coronavirus (MERS-CoV) and SARS-CoV-2 2,5,9. HCoV-229E, HCoV-OC43, HCoV-HKU1 and HCoV-NL63 usually cause mild symptoms, like common cold and/or diarrhea 10,11. In contrast, SARS-CoV, MERS-CoV and the newly-identified SARS-CoV-2 are highly pathogenic, causing severe lower respiratory tract infection in relatively more patients with a higher chance to develop acute respiratory distress syndrome (ARDS) and extrapulmonary manifestations.

The first HCoV-229E strain, B814, was isolated from the nasal discharge of patients with common cold in mid-1960s 12. Since then, more knowledge was accumulated through extensive studies on HCoV-229E and HCoV-OC43, both of which cause self-limiting symptoms 13. Indeed, the concept had been widely accepted that infection with HCoVs is generally harmless until the outbreak of SARS. The SARS outbreak occurred in 2003 is one of the most devastating in current history, infecting over 8,000 people with a crude case fatality of approximately 10% 14,15. Ten years later, the Middle East respiratory syndrome (MERS) outbreak resulted in a persistent epidemic in the Arabian Peninsula with sporadic spreading to the rest of the world 16-18. The 2019 novel HCoV (2019-nCoV), which has subsequently been renamed SARS-CoV-2, is the causative agent of the ongoing epidemic of coronavirus disease 2019 (COVID-19), which has claimed more than 3,120 lives and infected more than 91,000 people as of March 3, 2020 19. The alarm has been ringing and the world has to prepare for the coming pandemic of SARS-CoV-2.

All seven HCoVs have a zoonotic origin from bats, mice or domestic animals 2,20. Multiple lines of evidence support an evolutionary origin of all HCoVs from bats, where viruses are well adapted and non-pathogenic but show great genetic diversity. The COVID-19 epidemic has presented enormous medical, scientific, social and moral challenges to China and the world. Tracing the zoonotic origins of HCoVs provides a framework to understand the natural history, driving force and restriction factors of species jumping. This might also guide or facilitate the search for the reservoir, intermediate and amplifying animal host(s) of SARS-CoV-2, with important implications in the prevention of future spillovers. In this review we present an overview of the zoonotic origins, interspecies transmission and pathogenesis of HCoVs. Particularly, we highlight and discuss the common theme that parental viruses of HCoVs are typically non-pathogenic in their natural reservoir hosts but become pathogenic after interspecies transmission to a new host. We also review the trend of HCoV evolution in which the increase in transmissibility often comes with the decrease in pathogenicity. The outcome of the ongoing SARS-CoV-2 outbreak is also discussed in this context.

## History of HCoV discovery

Animal CoVs have been known since late 1930s. Before the first isolation of HCoV-229E strain B814 from the nasal discharge of patients who had contracted common cold, different CoVs had been isolated in various infected animals, including turkey, mouse, cow, pig, cat and dog 21-26. In the past decades, seven HCoVs have been identified. A brief summary of the history of HCoV discovery in chronological order (Table 1) would be informative and instructive.

### HCoV-229E and HCoV-OC43

The first HCoV-229E strain was isolated from the respiratory tract of patients with upper respiratory tract infection in the year of 1966 27, and was subsequently adapted to grow in WI-38 lung cell lines 28. Patients infected with HCoV-229E presented with common cold symptoms, including headache, sneezing, malaise and sore-throat, with fever and cough seen in 10~20% cases 29. Later in 1967, HCoV-OC43 was isolated from organ culture and subsequent serial passage in brains of suckling mice 28. The clinical features of HCoV-OC43 infection appear to be similar to those caused by HCoV-229E, which are symptomatically indistinguishable from infection with other respiratory tract pathogens such as influenza A viruses and rhinoviruses 28.

Both HCoV-229E and HCoV-OC43 are distributed globally, and they tend to be predominantly transmitted during the season of winter in temperate climate 2. Generally, the incubation time of these two viruses is less than one week, followed by an approximately 2-week illness 28. According to a human volunteer study, healthy individuals infected with HCoV-229E developed mild common cold 30. Only a few immunocompromised patients exhibited severe lower respiratory tract infection.

### SARS-CoV

SARS, also known as “atypical pneumonia”, was the first well documented HCoV-caused pandemic in human history and the etiological agent is SARS-CoV, the third HCoV discovered 14,15. The first case of SARS can be traced back to late 2002 in Guangdong Province of China. The SARS epidemic resulted in 8,096 reported cases with 774 deaths, spreading across many countries and continents. Apart from the super-spreaders, it was estimated that each case could give rise to approximately two secondary cases, with an incubation period of 4 to 7 days and the peak of viral load appearing on the 10^th^ day of illness 14,15.

Patients infected with SARS-CoV initially present with myalgia, headache, fever, malaise and chills, followed by dyspnea, cough and respiratory distress as late symptoms 14,15. Lymphopenia, deranged liver function tests, and elevated creatine kinase are common laboratory abnormalities of SARS 14,15. Diffuse alveolar damage, epithelial cell proliferation and an increase of macrophages are also observed in SARS patients 31. Approximately 20-30% of patients subsequently require intensive care and mechanical ventilation. In addition to lower respiratory tract, multiple organs including gastrointestinal tract, liver and kidney can also be infected in these severe cases, usually accompanied with a cytokine storm, which might be lethal particularly in immunocompromised patients. The virus was first isolated from the open lung biopsy of a relative of the index patient who travelled to Hong Kong from Guangzhou 14,15. Since then, tremendous efforts have been dedicated to HCoV research.

### HCoV-NL63 and HCoV-HKU1

HCoV-NL63 was isolated from a 7-month-old child from the Netherlands during late 2004. It was initially found to be prevalent in young children, the elderly and immunocompromised patients with respiratory illnesses 32. Presentation of coryza, conjunctivitis, fever, and bronchiolitis is common in the disease caused by HCoV-NL63 33. Another independent study described the isolation of the same virus from a nasal specimen from an 8-month-old boy suffering from pneumonia in the Netherlands 34. Although it was identified in Netherlands, it is actually distributed globally 2. It has been estimated that HCoV-NL63 accounts for approximately 4.7% of common respiratory diseases, and its peak incidence occurs during early summer, spring and winter 2. HCoV-NL63 is associated with obstructive laryngitis, also known as croup 35.

In the same year, HCoV-HKU1 was isolated from a 71-year-old man who had been hospitalized with pneumonia and bronchiolitis in Hong Kong 36. Besides community-acquired pneumonia and bronchiolitis, HCoV-HKU1 was reported to be associated with acute asthmatic exacerbation 37. Similar to HCoV-NL63, HCoV-229E and HCoV-OC43, HCoV-HKU1 was found worldwide, causing mild respiratory diseases 37. All these four community-acquired HCoVs have been well adapted to humans and are generally less likely to mutate to cause highly pathogenic diseases, though accidents did occur for unknown reasons as in the rare case of a more virulent subtype of HCoV-NL63, which has recently been reported to cause severe lower respiratory tract infection in China 38. Generally, when these HCoVs acquire the abilities to transmit efficiently and to maintain themselves continuously within humans, they also become less virulent or pathogenic.

### MERS-CoV

MERS-CoV was first isolated in 2012 from the lung of a 60-year-old patient who developed acute pneumonia and renal failure in Saudi Arabia 17,18,39. Whereas most of the laboratory-confirmed cases originate from the Middle East, imported cases with occasional secondary spreads to close contacts have been reported in various European countries and Tunisia. Another secondary outbreak occurred in South Korea in 2015 with 186 confirmed cases. Clinical manifestations of MERS resemble those of SARS, characterized by progressive acute pneumonia 17,18,39. Unlike SARS, many patients with MERS also developed acute renal failure, which is thus far unique for MERS among HCoV-caused diseases 17,18,39. More than 30% of patients present with gastrointestinal symptoms, such as diarrhea and vomiting 17,18,39. As of February 14, 2020, over 2500 laboratory confirmed cases were reported with a high case fatality of 34.4%, making MERS-CoV one of the most devastating viruses known to humans.

### SARS-CoV-2

During middle to late December 2019, clusters of pneumonia patients retrospectively known to be associated with SARS-CoV-2 infection were detected in Wuhan, Hubei Province, China 40. World Health Organization declared the ongoing outbreak of lower respiratory tract infection caused by SARS-CoV-2 a Public Health Emergency of International Concern and also named the disease COVID-19. As of March 3, 2020, 90,053 cases have been confirmed worldwide, with a crude case fatality of 3.4%. Notably, the case fatality in Hubei, China is 4.2%, whereas the one outside of it is 1.2%. SARS-CoV-2 causes severe respiratory infection like SARS-CoV and MERS-CoV, presented as fever, cough and dyspnea 40. Diarrhea is also seen in some patients 40. Pneumonia is one of the most severe symptoms and can progress rapidly to acute respiratory distress syndrome. Although SARS-CoV and SARS-CoV-2 are very similar due to high nucleotide sequence homology of 82%, they cluster into different branches in the phylogenetic tree 5. SARS-CoV-2 is apparently less pathogenic but more transmissible compared to SARS-CoV and MERS-CoV. Asymptomatic subjects infected with SARS-CoV-2 have been reported and might contribute to its rapid spreading around the world.

Comparing and contrasting SARS-CoV-2 with the other six HCoVs reveal similarities and differences of great interest. First, the incubation period and the duration of the course of HCoV disease are very similar. In this regard, SARS-CoV-2 follows the general trend of the other six HCoVs. Second, the severity of symptoms of COVID-19 lies between SARS-CoV and the four community-acquired HCoVs (i.e. HCoV-229E, HCoV-OC43, HCoV-HKU1 and HCoV-NL63). On one hand, SARS-CoV-2 infection exhibits features that are more commonly seen during infection with community-acquired HCoVs, including the presentation of non-specific, mild or even no symptoms. On the other hand, a small subset of severe cases of COVID-19 can also be seen as in the case of SARS-CoV infection, although the ratio is a bit lower. Third, the transmission of SARS-CoV-2 also shows interesting patterns characteristic of both community-acquired HCoVs and SARS-CoV. On one hand, the transmissibility of SARS-CoV-2 is at least as high as that of community-acquired HCoVs. On the other hand, it remains to be verified whether the transmissibility of SARS-CoV-2 decreases after passages in humans as in the cases of SARS-CoV and MERS-CoV. Finally, same as the other HCoVs 41, SARS-CoV-2 can be detected in fecal samples. Whether fecal-oral transmission of SARS-CoV-2 plays an important role as in the case of SARS-CoV at least under some circumstance remains to be clarified by future studies. It is also of particularly great interest to see whether SARS-CoV-2 might exhibit seasonality as in the cases of community-acquired HCoVs. Nevertheless, the features of SARS-CoV-2 including its transmissibility, pathogenicity and sustainable spreading after passages in humans will be influential on the ultimate fate of the ongoing outbreak of COVID-19.

## Animal origins of HCoVs

All four community-acquired HCoVs causing mild symptoms have been well adapted to humans. From another perspective, it might also be true that humans have been well adapted to these four HCoVs. In other words, both could be the survivors of ancient HCoV pandemics. HCoVs that cause severe diseases in humans and humans who developed severe HCoV diseases have been eliminated. For this to happen, HCoVs have to replicate in humans to sufficient extent to allow the accumulation of adaptive mutations that counteract host restriction factors. In this sense, the longer the SARS-CoV-2 outbreak persists and the more people that it infects, the greater chance that it will fully adapt to humans. If it adapts well, its transmission in humans would be difficult to stop by quarantine or other infection control measures.

For many years, the four community-acquired CoVs circulate in human populations, triggering common cold in immunocompetent subjects. These viruses do not need an animal reservoir. In contrast, highly pathogenic SARS-CoV and MERS-CoV have not adapted to humans well and their transmission within humans cannot be sustained. They need to maintain and propagate in their zoonotic reservoirs and seek the chance to spillover to susceptible human targets, possibly via one or more intermediate and amplifying hosts. SARS-CoV-2 has features that are similar to both SARS-CoV/MERS-CoV and the four community-acquired HCoVs. It is highly transmissible like community-acquired HCoVs, at least for the time being. However, it is more pathogenic than community-acquired HCoVs and less pathogenic than SARS-CoV or MERS-CoV. It remains to be seen whether it will adapt fully to humans and circulate within humans without a reservoir or intermediate animal host.

Before discussing the animal origins of HCoVs, it will serve us well to discuss the definitions and characteristics of evolutionary, natural, reservoir, intermediate and amplifying hosts of HCoVs. An animal serves as the evolutionary host of an HCoV if it harbours a closely related ancestor sharing high homology at the level of nucleotide sequence. The ancestral virus is usually well adapted and non-pathogenic in this host. Likewise, a reservoir host harbours HCoV continuously and for long term. In both cases, the hosts are naturally infected and are the natural hosts of HCoV or its parental virus. In contrast, if the HCoV is newly introduced to an intermediate host right before or around its introduction to humans, it is not well adapted to the new host and is often pathogenic. This intermediate host can serve as the zoonotic source of human infection and play the role of an amplifying host by allowing the virus to replicate transiently and then transmitting it to humans to amplify the scale of human infection. An HCoV can undergo a dead-end infection if it cannot sustain its transmission within the intermediate host. On the contrary, HCoVs can also adapt to the intermediate host and even establish long-term endemicity. In this case, the intermediate host becomes a natural reservoir host.

### SARS-CoV

Epidemiological data revealed retrospectively that the index case of SARS had a contact history with game animals 42. Subsequent seroprevalence investigations indicated that animal traders had a higher prevalence of anti-SARS-CoV IgG compared with that of the general population 42. Masked palm civets (*Paguma larvata*) and a racoon dog in live animal markets were first identified to carry SARS-CoV-like viruses that are almost identical to SARS-CoV 43. This was indirectly supported by the fact that no further SARS was reported after killing all civets in the markets. However, it has been reported that masked palm civets from the wild or farms without exposure to the live animal markets were largely negative for SARS-CoV 44, suggesting that masked palm civets might only serve as the intermediate amplifying host but not the natural reservoir of SARS-CoV. Notably, since 80% of the different animals in the markets in Guangzhou have anti-SARS-CoV antibodies 45, the possibilities that multiple species of small mammals might also serve as intermediate amplifying hosts of SARS-CoV cannot be excluded. All of these appear to be dead-end hosts of SARS-CoV.

Subsequent search for the natural animal host of SARS-CoV unveiled a closely related bat CoV, termed SARS-related *Rhinolophus* bat CoV HKU3 (SARSr-Rh-BatCoV HKU3), which exists in Chinese horseshoe bats 46. These bats are positive for anti-SARS-CoV antibodies and genome sequence of SARSr-Rh-BatCoV HKU3 37,47. This and other bat CoVs share 88-92% nucleotide sequence homology with SARS-CoV. These studies have laid the foundation for the new concept that bats host emerging human pathogens. Several SARS-like CoVs (SL-CoVs) have also been identified from bats, but none except for one designated WIV1 can be isolated as live virus 7. Human angiotensin converting enzyme 2 (ACE2) is known to be the receptor of SARS-CoV. WIV1 derived from fecal sample of bats was demonstrated to use bat, civet and human ACE2 as receptor for cell entry 48. Intriguingly, sera of convalescent SARS patients were capable of neutralizing WIV1 48. Thus far, WIV1 represents the most closely related ancestor of SARS-CoV in bats 7,48, sharing 95% nucleotide sequence homology. Albeit the high homology between these two viruses, it is generally believed that WIV1 is not the immediate parental virus of SARS-CoV and bats are not the immediate reservoir host of SARS-CoV.

### MERS-CoV

Phylogenetic analysis clusters MERS-CoV to the same group as bat CoV-HKU4 and bat CoV-HKU5. Bat CoV-HKU4 and MERS-CoV utilize the same host receptor, dipeptidyl peptidase 4 (DPP4), for virus entry 49. RNA-dependent RNA polymerase sequences of MERS-CoV are phylogenetically closer to counterparts in bat beta-CoVs identified from Europe and Africa 50,51. Up to now, no live MERS-CoV can be found in wild bats. MERS-CoV and its closest relative bat CoV-HKU25 share only 87% nucleotide sequence homology 52,53. Thus, bats might not be the immediate reservoir host of MERS-CoV. On the other hand, studies in Middle East have shown that dromedary camels are seropositive for MERS-CoV-specific neutralizing antibodies 54, same as camels of Middle East origin in multiple African countries 55. Live MERS-CoV identical to the virus found in humans was isolated from the nasal swabs of dromedary camels, further indicating that camels serve as the bona fide reservoir host of MERS-CoV 56. It is also noteworthy that generally mild symptoms but massive virus shedding were observed in camels experimentally infected with MERS-CoV 57. Notably, infected camels shed viruses not only through respiratory route but also through fecal-oral route, which is also the main route for virus shedding from bats. However, questions still remain since many confirmed cases of MERS have no contact history with camels prior to symptom onset 58, plausibly ascribed to human-to-human transmission or unknown transmission routes involving unrecognized animal species that harbour MERS-CoV. These merit further investigations.

### SARS-CoV-2

SARS-CoV-2 shares 96.2% nucleotide homology with a bat CoV RaTG13 isolated from *Rhinolophus affinis* bats 8. As in the cases of SARS-CoV and MERS-CoV, the sequence divergence between SARS-CoV-2 and RaTG13 is too great to assign parental relationship. That is to say, bats might not be the immediate reservoir host(s) of SARS-CoV-2 unless almost identical bat CoVs are found in future. Presumably, the intermediate animal hosts of SARS-CoV-2 should be among the wildlife species sold and killed at the Huanan Seafood Wholesale Market, with which many of the initial cases of COVID-19 were associated, indicative of a probable animal-to-human transmission event 40. Several recent studies based on metagenomic sequencing have suggested that a group of endangered small mammals known as pangolins (*Manis javanica*) could also harbour ancestral beta-CoVs related to SARS-CoV-2 59. These novel pangolin CoV genomes share 85-92% nucleotide sequence homology with SARS-CoV-2. However, they are equally closely related to RaTG13 with about 90% identity at the level of nucleotide sequence. They cluster into two sub-lineages of SARS-CoV-2-like viruses in the phylogenetic tree, one of which share a more similar receptor binding domain (RBD) with SARS-CoV-2, with 97.4% amino acid sequence identity 59. In stark contrast, the RBDs of SARS-CoV-2 and RaTG13 are more divergent, albeit a higher degree of sequence homology genome-wide. An earlier study on diseased pangolins also reported the detection of viral contigs from lung samples, which turn out to be similarly related to SARS-CoV-2 60. This study adopted different assembly methods and manual curation to generate a partial genome sequence comprising about 86.3% of the full-length viral genome 60.

We cannot exclude the possibility that pangolin is one of the intermediate animal hosts of SARS-CoV-2 59. However, currently there is no evidence in support of a direct pangolin origin of SARS-CoV-2 due to the sequence divergence between SARS-CoV-2 and pangolin SARS-CoV-2-related beta-CoVs. In addition, the distance between SARS-CoV-2 and RaTG13 is even shorter than that between SARS-CoV-2 and pangolin SARS-CoV-2-related beta-CoVs. The evolutionary pathway of SARS-CoV-2 in bats, pangolins and other mammals remains to be established. Whereas the highest sequence homology has been found in the RBDs between SARS-CoV-2 and pangolin, SARS-CoV-2-related beta-CoVs, SARS-CoV-2 and RaTG13 share the highest genome-wide sequence homology. It is highly speculative that the high degree of similarity between the RBDs of pangolin SARS-CoV-2-related beta-CoVs and SARS-CoV-2 is driven by selectivity-mediated convergent evolution. A counter-proposal is in favour of a recombination between a pangolin SARS-CoV-2-related beta-CoV and RaTG13 in the third wild animal species. As a driving force in evolution, recombination is widespread among beta-CoVs 61. The jury is still out on the immediate zoonotic origin of SARS-CoV-2.

### Community-acquired HCoVs

**Figure 1:**
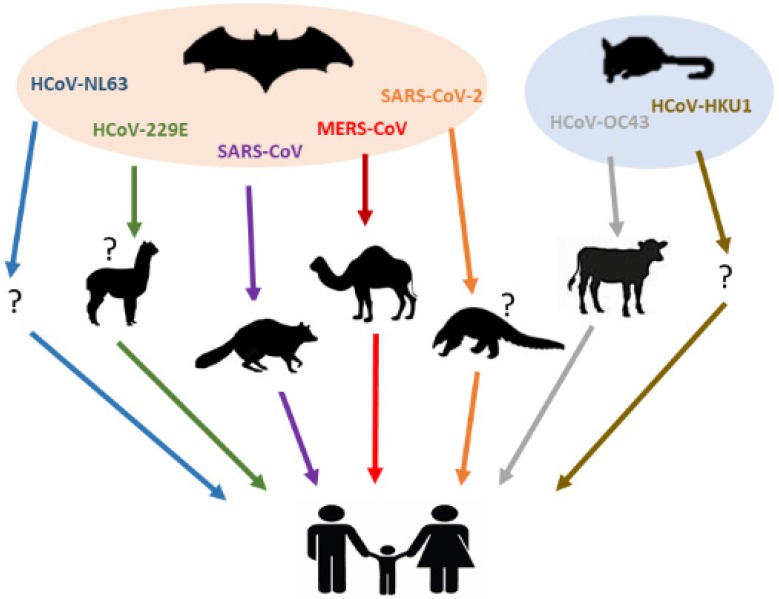
** Animal hosts of HCoVs.** Blue, green, purple, red, orange, grey, brown arrows represent the transmission of HCoV-NL63, HCoV-229E, SARS-CoV, MERS-CoV, SARS-CoV-2, HCoV-OC43 and HCoV-HKU1 from their natural hosts (bats or rodents) to the intermediate hosts (camelids, civets, dromedary camels, pangolins or bovines), and eventually to the human population. No concrete evidence exists on the intermediated host(s) of HCoV-NL63 and HCoV-HKU1, which was shown as a question mark (?).

Besides the highly pathogenic HCoVs, the zoonotic origin of HCoV-229E, HCoV-OC43, HCoV-NL63 and HCoV-HKU1 have also been studied 9,62-64. Phylogenetic evidence indicated that both HCoV-NL63 and HCoV-229E might have originated from bat CoVs 62-64, while the parental viruses of HCoV-OC43 and HCoV-HKU1 have been found in rodents 9. It has been reported that a bat CoV termed ARCoV.2 (Appalachian Ridge CoV) detected in North American tricolored bat displayed close relationship with HCoV-NL63 62,63. On the other hand, HCoV-229E was genetically related to another bat CoV, termed Hipposideros/GhanaKwam/19/2008, which was detected in Ghana 65, while camelids have also been suspected as its intermediate host 66,67. For clarity, the current knowledge on animal origins of known HCoVs is summarized in Figure 1 and Table 2.

## Interspecies transmission of HCoVs

Phylogenetic analysis has provided evidence for interspecies transmission events of HCoVs in the history. When HCoV-OC43 crossed species to infect humans from domestic livestock around 1890, a pandemic of respiratory infection was recorded 68. The interspecies transmission history of HCoV-229E is less clear. Bat alpha-CoVs closely related to HCoV-229E have been found. Between them there is an alpaca alpha-CoV. Several lines of evidence support the transmission of virus from bats to humans directly. First, humans but not alpacas might have contact with bats in a shared ecological niche. Instead, humans have close contact with alpacas. Second, HCoV-229E-related bat alpha-CoVs are diverse and non-pathogenic in bats, whereas alpaca alpha-CoV caused an outbreak of respiratory disease in infected animals 65. Finally, alpaca alpha-CoV has not been found in feral animals. Thus, the possibility cannot be excluded that alpacas obtain the HCoV-229E-related alpha-CoV from humans. In fact, bats are the direct source of human pathogenic viruses including rabies virus, Ebola virus, Nipah virus and Hendra virus 69. It is therefore not too surprising that bats might transmit HCoV-229E to humans directly. Alternatively, whereas bat alpha-CoVs serve as the gene pool of HCoV-229E, alpacas and dromedary camels might serve as intermediate hosts that transmit viruses to humans, exactly as in the case of MERS-CoV 69.

### Host determinants of transmission

MERS-CoV serves as an excellent example of interspecies transmission from bats to dromedary camels and from dromedary camels to humans. The evolutionary origin of MERS-CoV from bats is known at its initial identification and has also been strengthened by subsequent findings 49-51. It is obvious that bats provide a rich pool of virus species for interspecies exchange of genetic fragments and interspecies transmission. Longevity, densely packed colonies, close social interaction and strong ability to fly are all favourable conditions for bats to be an ideal 'virus spreader'. On the other hand, MERS-CoV has been introduced to dromedary camels for decades. It is well adapted to these camels that have turned from an intermediate host to a stable and natural reservoir host. MERS-CoV causes very mild disease and maintains a relatively low mutation rate in these animals. Its sporadic transmission to humans is an accident and humans remain a dead-end host of MERS-CoV as its transmission cannot be sustained.

In contrast to the role of camels in the transmission of MERS-CoV, the role of pangolins, if there is any, in the transmission of SARS-CoV-2 is different. Particularly, pangolin beta-CoVs are highly pathogenic in pangolins. They might be a dead-end host for SARS-CoV-2-related beta-CoVs, similar to civets in the case of SARS-CoV. Several possibilities for interspecies transmission of SARS-CoV-2 from animals to humans have to be ruled in or ruled out in future studies. First, bats could be the reservoir host of a SARS-CoV-2-related virus almost identical to SARS-CoV-2. Humans might share the ecological niche with bats through butchering or coal mining. Second, pangolins could be one of intermediate amplifying host to which a SARS-CoV-2-related virus had been newly introduced. Humans contract the virus through butchering and consumption of game meat. It is possible that many mammals including domestic animals are susceptible to SARS-CoV-2. A survey of domestic and wild animals for antibodies is warranted. Third, as mentioned above, recombination and adaptation of SARS-CoV-2 might have occurred in a third species that has contact with both bats and pangolins. The search for the animal origins of SARS-CoV-2 is still on.

### Viral determinants of transmission

Apart from different types of the animal hosts, three major factors on the viral side are also important in facilitating CoVs to cross species barriers 70. First of all, their relatively high mutation rates in RNA replication. In comparison to other single-stranded RNA viruses, the estimated mutation rates of CoVs could be regarded as “moderate” to “high” with an average substitution rate being ~10^-4^ substitution per year per site 2, depending on the phase of CoV adaptation to novel hosts. CoVs have a proof-reading exoribonuclease, deletion of which results in exceedingly high mutability and attenuation or even inviability. Interestingly, the nucleotide analogue Remdesivir is known to suppress CoV replication through inhibition of this exoribonuclease and the RNA-dependent RNA polymerase 70. Remdesivir is one of most promising anti-SARS-CoV-2 agents to be tested in clinical trials. Nevertheless, mutation rates of CoVs are about a million times higher than those of their hosts. In addition, mutation rate is often high when CoVs are not well adapted to the host 71. Compared to SARS-CoV with a high mutation rate 72, the mutation rate of SARS-CoV-2 is apparently lower, suggestive of a higher level of adaptation to humans. Presumably, it has already been adapted to another host close to humans. In addition to SARS-CoV-2, this also applies to MERS-CoV, which is well adapted to dromedary camels. Theoretically, it is unlikely that genetic drift would render vaccines and antivirals against SARS-CoV-2 ineffective quickly. Second, the large RNA genome in CoVs exerts extra plasticity in genome modification for mutations and recombination, thereby increasing the probability for interspecies co-evolution, which is advantageous for the emergence of novel CoVs when the conditions become appropriate. This is supported by the copious unique open reading frames and protein functions encoded towards the 3′ end of the genome. Third, CoVs randomly and frequently switch templates during RNA replication through a unique “copy-choice” mechanism. In a host that serves as the mixing vessel, strand switching occurs frequently during CoV RNA transcription. Highly homologous full-length and subgenomic RNAs could recombine to generate new CoVs. Phylogenetic evidence of natural recombination has been found in both HCoV-HKU1 and HCoV-OC43, as well as animal CoVs such as bat SL-CoV and batCoV-HKU9 73.

### Virus-host interaction in relation to transmission

Besides three viral factors stated above, viral interaction with host receptor is another key factor influential on interspecies transmission. Herein, recombination of SARS-CoV is taken as a typical example, which also showed evidence of positive selection during interspecies transmission events 74. Based on the comparative analysis between isolates of human and civet SARS-CoVs, SARS-CoV is thought to undergo rapid adaptation in different hosts, particularly with mutations at the RBD of the S protein 74. Generally, the RBD in the S protein of a CoV interacts with the cellular receptor and is intensely selected by the host antibody response. In SARS-CoV, the RBD is in the 318^th^ to 510^th^ amino acids on the S1 fragment, which binds to the human ACE2 as well as its coreceptors for viral entry 75. The RBD of SARS-CoV is capable of recognizing the ACE2 receptors of various animals, including bat, civet, mouse and raccoon dog, allowing interspecies transmission of the virus. In fact, only 6 amino acid residues were observed to be different from human and civet viral isolates in the RBD and 4 of them locate in the receptor-binding motif for interaction with the ACE2 receptor. Civet SARS-CoV has K479N and S487T mutations in its RBD, which might increase the affinity of the interaction of spike protein with human ACE2 receptor. In other words, these two amino acid substitutions might be critical to viral adaption to humans 75.

It is noteworthy that SARS-CoV-2 shares the same cellular receptor with SARS-CoV 8. A 30% difference between SARS-CoV-2 and SARS-CoV in the S1 unit of the S protein implicates that the binding affinity of its S protein with human ACE2 might have altered 5. Indeed, a cryo-EM study indicates a 10- to 20-fold higher affinity of this binding than that between human ACE2 and SARS-CoV S protein 76. It will also be of interest to determine whether any other coreceptor might be required for SARS-CoV-2 transmission. Intriguingly, HCoV-NL63 also binds to ACE2 but with a different part of S 77. There exist many other HCoV receptors, such as aminopeptidase N for HCoV-229E, and 9-O-acetylated sialic acid for HCoV-OC43. They might also account for successful adaptation of these CoVs in humans after interspecies transmission from their animal hosts.

In addition to cellular receptors, the outcome of interspecies transmission of HCoVs is also governed by other host dependency and restriction factors. The divergence of these host proteins between humans and natural reservoir hosts of HCoVs such as bats, dromedary camels and rodents might constitute a barrier to interspecies transmission. HCoVs have to usurp host dependency factors and subvert host restriction factors for a successful interspecies transmission. In this regard, molecular determinants in this important area of virus-host interaction remain to be identified and characterized. An unbiased genome-wide screening of host dependency and restriction factors for SARS-CoV-2 using the state-of-the-art technology of CRISPR might be fruitful.

### Emergence of novel HCoVs: back to ground zero

The diversity of bat CoVs provides ample opportunities for the emergence of novel HCoVs. In this sense, bat CoVs serve as the gene pool of HCoVs. In addition, rapid mutation and genetic recombination also drive HCoV evolution and serve as two important steps in this process 2,6. For example, the acquisition or loss of novel protein-coding genes has the potential to drastically modify viral phenotypes. Among SARS-CoV accessory proteins, ORF8 has been thought to be important in adaptation to humans, as SARS-CoV-related bat viruses were isolated but found to encode divergent ORF8 proteins 48. A 29-nucleotide deletion characteristic of SARS-CoVs has been found in strains isolated at the beginning of the human epidemic. This deletion splits ORF8 into ORF8a and ORF8b and is thought to be an adaptive mutation that promotes the switch of hosts 78. Besides, SARS-CoV has a possible recombination history with lineages of alpha- and gamma-CoVs, where a large number of smaller recombinant regions were identified in the RNA-dependent RNA polymerase 72. Recombination locations were also identified in the nsp9, most of nsp10, and parts of nsp14 72. Likewise, it has been shown that the epidemic MERS-CoV experienced recombination events between different lineages, which occurred in dromedary camels in Saudi Arabia 79. Besides SARS-CoV and MERS-CoV, recombination events have also been observed in other HCoVs, in which the HCoVs recombine with other animal CoVs in their non-structural genes.

It should also be cautioned that artificial selection can contribute to unintended changes in viral genomes, most likely resulting from relieving viruses from selection pressures exerted, such as by the host immune system. An example of these effects is the loss of a full-length ORF4 in the HCoV-229E prototype strain, owing to a two-nucleotide deletion 80. While intact ORF4 could be observed in bat and camel viruses related to HCoV-229E, the alpaca alpha-CoV displays a single nucleotide insertion, resulting in a frameshift 81.

Last but not least, the evolution of novel HCoVs is also driven by the selection pressure in their reservoir hosts. Asymptomatic or only mild symptoms were detected when bats were infected with CoVs, indicating the mutual adaptation between CoVs and bats 82. It appeared that bats are well adapted to CoVs anatomically and physiologically. For example, defects in the activation of pro-inflammatory response in bats efficiently reduce the pathology triggered by CoVs 83. Besides, the natural killer cell activity in bats is suppressed due to upregulation of inhibitory natural killer cell receptor NKG2/CD94 and low expression level of major histocompatibility complex class I molecules 84. Moreover, the high level of reactive oxygen species (ROS) generated from high metabolic activity of bats could both suppress CoV replication and affects proofreading by exoribonuclease 85, thus providing the selection pressure for the generation of virus strains highly pathogenic when introduced into a new host. More pathogenic CoV strains might also evolve by recombination, leading to the acquisition of novel proteins or protein features for host adaptation 82. Thus, it is not by chance that three novel HCoVs have emerged in the past two decades.

CoVs are non-pathogenic or cause mild symptoms in their reservoir hosts such as bats and camels. They replicate robustly without eliciting a strong host immune response. Herein lie the secrets of why asymptomatic carriers are seen and what causes the severe cases in human infection. The severe symptoms are mainly due to the hyperactivation of immune response and the cytokine storm wherein the stronger the immune response, the more severe the lung damage. In contrast, in asymptomatic carriers, the immune response has been de-coupled from CoV replication. The same strategy of delinking the immune response might have beneficial effects in anti-SARS-CoV-2 therapy. The interferon response is particularly strong in bats. Thus, administration of type I interferon at least in the early phase of SARS-CoV-2 infection in humans should be beneficial. In addition, NLRP3 inflammasome activation in bats is defective 86. By this reasoning, inhibition of NLRP3 inflammasome with MCC950 might be useful in the treatment of COVID-19.

The emergence of SARS-CoV-2 follows the general theme by which SARS-CoV and MERS-CoV arose. Whereas a bat beta-CoV sharing 95% nucleotide homology with SARS-CoV has been found, there also exists a bat-CoV sharing 96% nucleotide homology with SARS-CoV-2. Whereas civets and other animals in the markets have been found to harbour viruses identical to SARS-CoV, immediate intermediate hosts for SARS-CoV-2 have not been identified. Pangolin beta-CoVs strikingly homologous to SARS-CoV-2 have been found, indicating that pangolins might serve as one of intermediate hosts or pangolin beta-CoVs could contribute gene fragments to the final version of SARS-CoV-2. Although questions remain, there is no evidence that SARS-CoV-2 is man-made either deliberately or accidentally.

## Conclusions

CoVs have returned to the limelight due to the recent outbreak of SARS-CoV-2. The study of CoVs in bats and other animals has drastically changed our perception of the importance of zoonotic origins and animal reservoirs of HCoVs in human transmission. Pervasive evidence has shown that SARS-CoV, MERS-CoV and SARS-CoV-2 have a bat origin and are transmitted to humans via intermediate hosts. Given that SARS-CoV infection originates from the contact between humans and civets in the markets, closing wet markets and killing civets therein could have effectively ended the SARS epidemic. By the same reasoning, pangolins should be removed from wet markets to prevent zoonotic transmission, in view of the discovery of multiple lineages of pangolin beta-CoVs closely related to SARS-CoV-2. However, whether and how SARS-CoV-2 is transmitted to humans through pangolins and other mammals remain to be clarified in future investigations.

On the other hand, MERS-CoV has existed in dromedary camels for a long time. These camels serve as an important tool for transportation as well as a main source of meat, milk, leather and wool products for the local people. They are widely distributed across the Middle East and Africa. It is therefore impossible to sacrifice all camels for the control of MERS, as what was done in wild animal markets in China to prevent the spreading of SARS-CoV and SARS-CoV-2. To stop the recurrent outbreaks of MERS, a comprehensive approach should be taken to develop effective vaccines against MERS-CoV for camels, in combination with other infection control measures. As we are not able to eliminate these viruses, new genotypes might emerge to cause outbreaks.

A variety of zoonotic CoVs are circulating in the wild. Particularly, bat CoVs with zoonotic potential are so diverse. There are plenty of opportunities that these zoonotic CoVs evolve and recombine, resulting in the emergence of new CoVs that are more transmissible and/or deadly in humans in future. The culture of eating wild animals in some places of China should be abandoned to reduce unnecessary contact between humans and animals. With the ordeals of SARS, MERS and COVID-19, a better preparedness and response plan should be in place. In fact, many viruses have existed in the planet for a very long time. They stay in their own natural reservoirs until there is a chance for spillover. Although bats have many features that favours the spreading of viruses, the chance for humans to be in contact with bats and other wildlife species can be minimized if people are educated to stay away from them. Continuous surveillance in mammals is necessary for better understanding of the ecology of CoVs and their natural hosts, which will prove useful in preventing animal-to-human transmission and future outbreaks. To conclude, the most effective way to prevent viral zoonosis is for humans to stay away from the ecological niches of the natural reservoirs of the zoonotic viruses.

Several pieces in the puzzle of the zoonotic origin of SARS-CoV-2 are still missing. First, if bats transmit an ancestral virus of SARS-CoV-2 to pangolins, it will be of interest to see under what circumstances bats and pangolins could share the same ecological niche. Second, if bats play a more direct role in human transmission, how humans get into contact with bats should be determined. Third, if a third mammal acts as the true intermediate host, how it interacts with the different species including humans, bats and pangolins has to be clarified. Finally, since many mammals including domestic animals might be susceptible to SARS-CoV-2, both surveillance and experimental infection should be conducted. Should it be a bat, a pangolin or another mammal, it is expected that SARS-CoV-2 or its parental viruses that are almost identical will be identified in its natural hosts in future. Continued investigations in this area will elucidate the evolutionary pathway of SARS-CoV-2 in animals, with important implications in the prevention and control of COVID-19 in humans.

## Figures and Tables

**Table 1:** Comparison of clinical features and transmission routes of HCoVs

	HCoV-229E	HCoV-OC43	SARS-CoV	HCoV-NL63	HCoV-HKU1	MERS-CoV	SARS-CoV-2
Classification	alpha-CoV	beta-CoV, lineage A	beta-CoV, lineage B	alpha-CoV	beta-CoV, lineage A	beta-CoV, lineage C	beta-CoV, lineage B
Incubation period	2-5 days	2-5 days	2-11 days	2-4 days	2-4 days	2-13 days	3-6 days
Transmission	Respiratory droplets Fomites	Respiratory droplets Fomites	Respiratory droplets Fomites Fecal-oral	Respiratory droplets Fomites	Respiratory droplets Fomites	Respiratory droplets Fomites	Respiratory droplets Fomites Fecal-oral
Case fatality	N/A	N/A	9.6%	N/A	N/A	34.4%	3.5%
Clinical symptoms	Malaise Headache Nasal discharge Sneezing Sore throat Fever and cough	Malaise Headache Nasal discharge Sneezing Sore throat Fever and cough	Fever Myalgia Headache Malaise Dry cough Dyspnea Respiratory distress Diarrhea	Cough Rhinorrhea Tachypnea Fever Hypoxia Croup	Fever Running nose Cough Dyspnea	Fever Cough Chills Sore throat Myalgia Arthralgia Dyspnea Pneumonia Diarrhea and vomiting Acute renal impairment	Fever Dry cough Dyspnea Myalgia Headache Diarrhea
Epidemiology	Globally Peak in winter	Globally Peak in winter	2002-2003 in China Globally thereafter	Globally Peak in winter	Globally Peak in winter	2012 in Middle East 2015 in South Korea Endemic in Middle East	2019-2020 in China Globally thereafter
References	27-30	28	14, 15, 31	32-35	36, 37	17, 18, 39	40

**Table 2:** Animal origins of HCoVs

HCoV	Natural host	Intermediate host	References
HCoV-229E	Bats	Camelids?	65-67
HCoV-OC43	Rodents	Bovines	9
SARS-CoV	Bats	Palm civets	7, 37, 42-48
HCoV-NL63	Bats	Unidentified	62, 63
HCoV-HKU1	Rodents	Unidentified	9
MERS-CoV	Bats	Dromedary camels	49-58
SARS-CoV-2	Bats	Pangolins?	8, 59

## Abbreviations

SARS: Severe acute respiratory syndrome
MERS: Middle East respiratory syndrome
COVID-19: Coronavirus disease 19
SARS-CoV: Severe acute respiratory syndrome coronavirus
MERS-CoV: Middle East respiratory syndrome-CoV
ORF: Open reading frame
ACE2: Angiotensin-converting enzyme 2
DPP4: Dipeptidyl peptidase 4
SL-CoV: SARS-like coronavirus
